# Quercetin reshapes gut microbiota homeostasis and modulates brain metabolic profile to regulate depression-like behaviors induced by CUMS in rats

**DOI:** 10.3389/fphar.2024.1362464

**Published:** 2024-03-26

**Authors:** Bozhi Li, Yuqi Yan, Tiange Zhang, Hanfang Xu, Xiaofeng Wu, Gaolei Yao, Xingze Li, Can Yan, Li-Li Wu

**Affiliations:** Integrative Medicine Research Center, School of Basic Medical Sciences, Guangzhou University of Chinese Medicine, Guangzhou, China

**Keywords:** depression, quercetin, brain metabolomics, gut microbiota, microbiota-gut-brain axis

## Abstract

Quercetin, an abundant flavonoid compound in plants, is considered a novel antidepressant; however, its mechanisms of action are poorly understood. This study aimed to investigate the therapeutic effects of quercetin on chronic unpredictable mild stress (CUMS)-induced depression-like behaviors in rats and explore the underlying mechanisms by combining untargeted metabolomics and 16S rRNA sequencing analysis of brain tissue metabolites and gut microbiota. Gut microbiota analysis revealed that at the phylum level, quercetin reduced *Firmicutes* and the *Firmicutes/Bacteroidetes (F/B)* ratio and enhanced *Cyanobacteria*. At the genus level, quercetin downregulated 6 and upregulated 14 bacterial species. Metabolomics analysis revealed that quercetin regulated multiple metabolic pathways, including glycolysis/gluconeogenesis, sphingolipid metabolism, the pentose phosphate pathway, and coenzyme A biosynthesis. This modulation leads to improvements in depression-like phenotypes, anxiety-like phenotypes, and cognitive function, highlighting the therapeutic potential of quercetin in treating depression.

## Introduction

Depression is a complex mental disorder that causes 10% of global disability. Its primary symptoms include low mood, anxiety, anhedonia, and cognitive impairment (Cruz-Pereira et al., 2020). Recently, notably during the COVID-19 pandemic, the incidence and mortality rates of depression have continuously increased, becoming an increasingly heavy burden on individuals and society. Depression is linked to reductions in brain monoamine neurotransmitters (serotonin, norepinephrine, and dopamine), changes in brain neurotrophic factor levels, abnormal activation of the hypothalamic-pituitary-adrenal axis, and immune system dysregulation. In addition to these factors, many recent studies have found that abnormalities in the gut microbiota are significant factors in developing depression (Jiang et al., 2015; Averina et al., 2020; Huang and Wu, 2021). The gut, known as the “second brain,” can regulate human emotions and feelings through the gut-brain axis. The gut microbiota, known as the “second genome” of the body, functions in symbiosis with the host and has major impacts. Therefore, regulating the gut microbiota is an innovative therapy for complex central nervous system (CNS) disorders, although the precise mechanism of the brain-gut axis remains to be elucidated.

Clinical depression treatment in clinical practice mainly relies on medication, with numerous antidepressants applied since the introduction of the first antidepressant in the 1950s (Hammen, 2018). However, these antidepressant drugs often fail to alleviate depression symptoms completely and may lead to severe drug dependence and side effects (Bschor et al., 2014; Solem et al., 2017). Plant-derived compounds are being evaluated for clinical depression treatment. Quercetin is a neuroprotective flavonoid found in flowers, leaves, and fruits. It is primarily transformed in the gut by the gut microbiota and absorbed by the human body (Russo et al., 2012). Research has revealed that quercetin can improve gut microbiota dysbiosis, promote gut microbial balance, restore gut barrier structure and function, and exhibit biological activities such as anti-inflammatory, antioxidant, and antiviral effects (Horowitz and Zunszain, 2015; Suganthy et al., 2016; Babaei et al., 2018; Ulusoy and Sanlier, 2020). It also demonstrates potential pharmacological activities against mental disorders (Chen et al., 2022). Moreover, compared to traditional antidepressants, quercetin has several advantages: 1) Multiple mechanisms of action: Numerous studies have reported that quercetin may exert its antidepressant effects via various mechanisms, such as antioxidation, anti-inflammation, neuroprotection, and modulation of the neurotransmitter system (Sah et al., 2011; Yang et al., 2020; Zhang et al., 2020; Şahin et al., 2020; Ma et al., 2021); 2) Fewer side effects and non-addictive: Quercetin is associated with fewer side effects and lacks the addictive potential of traditional antidepressants, which are often linked to significant side effects (Bschor et al., 2014; Solem et al., 2017). As a natural compound, quercetin is generally considered safer, has milder side effects, and there are no reported cases of addiction, making it potentially a safer option for long-term use (Kashyap et al., 2019; Ulusoy and Sanlier, 2020); 3) Additional health benefits: In addition to its potential antidepressant properties, quercetin offers a range of other health advantages, such as lowering the risk of cardiovascular diseases, exhibiting anticancer effects, and boosting immune function (Li et al., 2016; Rauf et al., 2018; Hosseini et al., 2021). However, how quercetin modulates the “microbiota-gut-brain” axis to treat depression remains unknown. Consequently, it is essential to further investigate the regulatory effects of quercetin on the brain and gut microbiota and explore its antidepressant mechanism.

**FIGURE 1:**
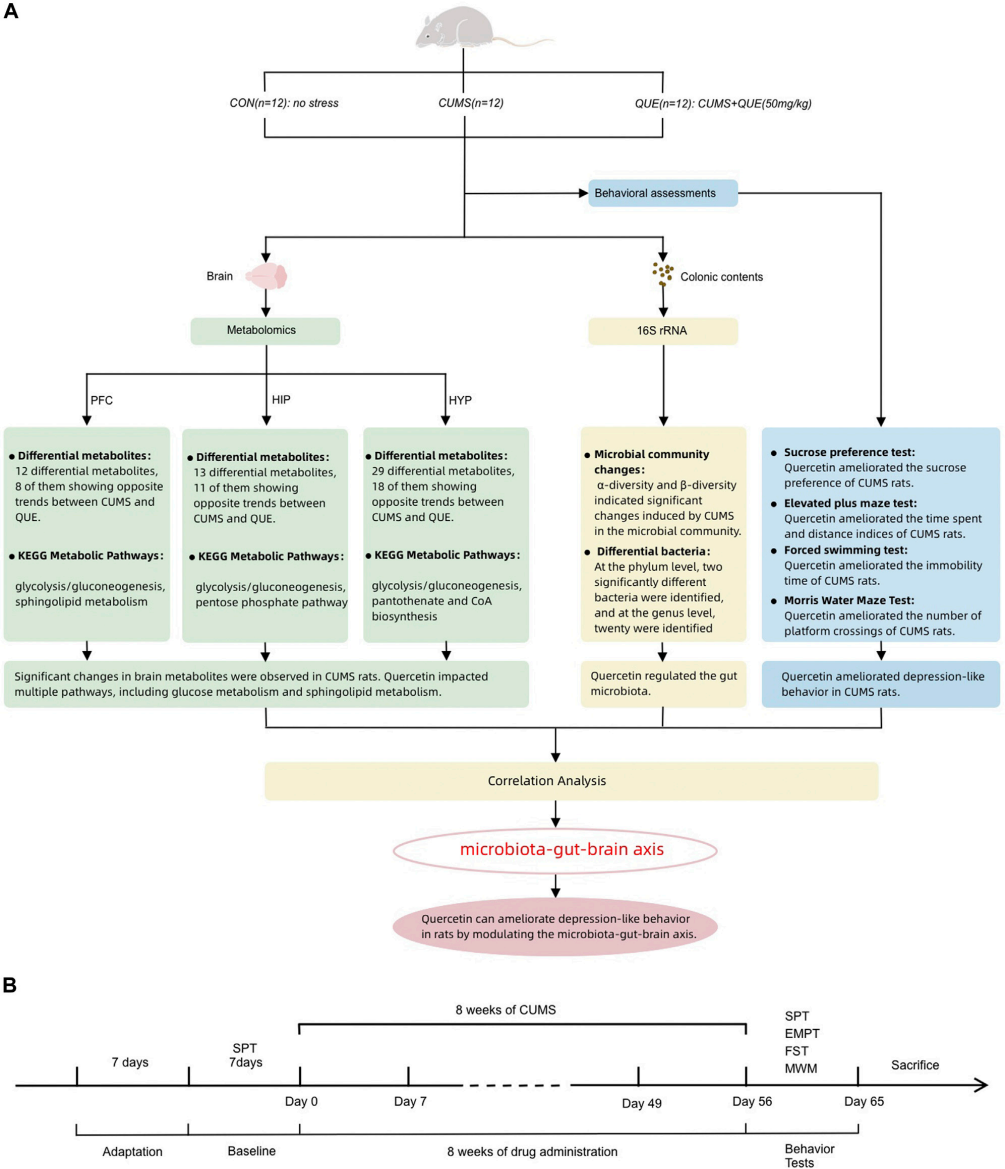
Summary of experimental procedure and results **(A)** Overview of behavioral analysis, gut microbiota analysis, and brain metabolism (prefrontal cortex, hippocampus, and hypothalamus) analysis in the CON, CUMS, and QUE groups of rats. CON, Control; QUE, Quercetin; CUMS, Chronic Unpredictable Mild Stress; SPT, Sucrose Preference Test; EPMT, Elevated Plus-Maze Test; FST, Forced Swim Test; MWM, Morris WaterMaze. **(B)** Experimental rat modeling and behavioral test workflow.

Metabolomics is a novel technology that utilizes various modern analytical techniques to determine dynamic changes in small-molecule metabolites in biological organisms, thereby characterizing and deciphering the status of life activities. It can more accurately and directly reflect the terminal and phenotypic information of biological systems, providing a new perspective for understanding the multi-factor mechanisms of diseases and comprehensively evaluating drug effects (Gu and Tong, 2020). Herein, 16S rRNA full-length sequencing and liquid chromatography-mass spectrometry (LC-MS) were employed to conduct untargeted metabolomic analysis of brain tissue metabolites and microbiota analysis of gut microbiota in rats to investigate the mechanism by which quercetin acts on the microbiota–gut–brain axis to regulate gut microbiota and brain metabolism and alleviate depression (Figure 1A).

## Materials and methods

### Experimental animals

Adult male Wistar rats (weighing 180–220 g) with specific pathogen-free (SPF) status were acquired from the Experimental Animal Center of the Southern Medical University in Guangzhou, China (license number: SCXK 2016-0041). All experiments involving animals adhered to the guidelines outlined in the “Regulations on the Management of Laboratory Animals” issued by the Ministry of Science and Technology of the People’s Republic of China. Ethical approval for this research was obtained from the Animal Experiment Ethics Committee of Guangzhou University of Chinese Medicine.

### Animal model

Prior to the commencement of the experiment, the rats were subjected to a 1-week acclimatization period in a controlled environment. This environment was maintained under a 12-h light/dark cycle, at a constant temperature of 23°C ± 2°C and a relative humidity of 60% ± 5%. To mitigate stress responses, rats were allowed unrestricted access to food and water. Subsequently, all rats underwent the sucrose preference (SP) test (SPT) to determine their individual responsiveness. Rats with aberrant baseline sucrose consumption were systematically removed from further analyses based on specific criteria. These criteria included low (<60%) SP, positional preference (showing a pronounced inclination to drink from a specific location), minimal water intake (indicating a lack of preference between sucrose solution and pure water), and excessive water intake (total water consumption exceeding twice the mean water intake of all rats). All rats were housed individually throughout the SPT to ensure isolation and accurate data collection. After SPT, the rats were randomly allocated to the control (CON), model (chronic unpredictable mild stress [CUMS]), and quercetin (QUE) groups, each consisting of 12 rats. The rats in the CON group were provided unrestricted access to both food and water, adhering to a 12-h light/dark cycle where the lights were illuminated from 8:00 to 20:00). Furthermore, the rats in the CON group were maintained at a temperature of 23°C ± 2°C, and any supplementary stressors were intentionally eliminated from the experimental conditions experienced by the rats. These rats were housed in cages with four rats per cage and accommodated in Room A to facilitate breeding. However, the rats belonging to the experimental groups, excluding the CON group, were exposed to the 12 stressors constituting the CUMS protocol. To ensure individualized housing, each rat was placed in a separate cage and subsequently transferred to Room B. The stressors included in the experimental design involved various challenges. These conditions included food deprivation (12 h), water deprivation (12 h), simultaneous deprivation of both food and water (24 h), imposition of restraint stress (12 h), exposure to damp bedding (10 h), engagement in overcrowded conditions (10 h), exposure to overnight intermittent light (300 times/min, 5 h), white noise exposure (85 dB, 5 h), engagement in thermal swimming (45°C, 5 min), engagement in cold swimming (4°C, 5 min), exposure to foot shocks (1 mA, 2 s per trial, ten trials in 5 min), and continuous light exposure during the night (20:00 to 8:00, 12 h). The stressors were randomly administered as 1-2 types daily, without repetition, within 3 days and continued for 8 weeks. The entire stress process is illustrated in Figure 1B.

### Administration method

From the day the stress application commenced, the rats in each group received intragastric administration for 8 weeks and ceased when the stress application was terminated. Rats in CON and CUMS groups received a daily dose of 5 mL/kg of pure water via intragastric administration. In contrast, rats in the QUE group received a daily dose of 50 mg/kg quercetin via intragastric administration.

### Behavioral tests

#### SPT

The SPT test was utilized to assess anhedonia, a key manifestation of depression-like behaviors, by examining the preference of rats for sucrose solution *versus* water. To maintain controlled conditions, all rats were individually housed during the experiment. The SPT involved four phases: 48-h sucrose training, 36-h baseline test, 24-h phase of food and water deprivation, and 12-h session dedicated to the actual SPT. In the final phase of the experiment, the rats were given unrestricted access to equal volumes of pure water and 1% sucrose solution for 12 h. The calculation of basic SP was conducted using the following formula: sucrose consumption/total liquid consumption × 100%. To assess SP, an SPT was performed in the model group after an 8-week duration. The model group was subjected to SPT for 12 h after a 24-h period of food and water deprivation.

#### Elevated plus-maze test

The EPMT is a critical tool for assessing anxiety-like behaviors in rodents. This methodology involved measuring the duration of stay and the number of entries into the open and closed arms of the maze, thereby facilitating a detailed analysis of anxiety-related behaviors. The maze was composed of a central area measuring 15 × 15 cm, from which two open arms measuring 15 × 48 cm and two closed arms measuring 15 × 48 × 40 cm. The experimental setup involved raising the apparatus to a height of 36 cm above the ground. For the testing process, the rat was positioned in a specific orientation to face the designated side of the maze. The open arms, positioned in the central area, were the focus of the test. The movements of the rats were recorded for 5 min using a camera. Multiple parameters were systematically measured to assess the anxiety-like levels exhibited by each rat. These parameters consisted of the following: 1) the time spent and percentage of time spent in the open arms, indicating the duration of the rat stay in the open arms and the percentage of the total testing period allocated to the open arms; 2) the distance traveled and percentage of the distance covered in open arms, denoting the total distance covered by the rat along with the percentage of that distance covered in the open arms; 3) the number of entries and percentage of entries made by the rat into the open arms, representing the total number of entries made by the rat into the open arms and the corresponding percentage of total entries during the test.

#### Forced swimming test

The FST was used to evaluate depression-like behaviors in rats by measuring their immobility time in water. The test was conducted in a transparent cylindrical tank as the swimming chamber and was characterized by the following dimensions: 30 cm in diameter, 100 cm in height, and a water depth of 35 cm. To ensure consistency, the water temperature was maintained at 25°C ± 1°C. To facilitate acclimatization to the laboratory environment, the rats were positioned in the apparatus room for 1 h before the experiment. The order of testing was randomized for each group. During the test, the experimenter gently held the rat by the tail, approximately two-thirds of the base, and slowly placed it into the swimming chamber. The behavior of each rat was recorded using a camera for 6 min. Subsequently, the experimenters, who were unaware of the group assignments, manually recorded the immobility time of each rat during the last 5 min of the test. Immobility manifests when a rat assumes a floating position on the water surface, displaying a lack of limb movement or subtle paddling motions with its forepaws and tail to sustain the head above the water.

#### Morris water maze test

The evaluation of spatial learning and memory in rats was conducted by applying the MWM test. This experimental approach involved quantifying the time and path chosen by the rats while navigating through a water maze to locate a hidden platform. The test had two stages: the acquisition navigation trial and the spatial probe trial, which lasted 6 days at 25°C. The water pool was uniformly partitioned into four quadrants, and the platform was strategically positioned at the center of one of the quadrants. The camera system displayed above the maze recorded rat movement trajectories synchronously. The acquisition navigation test was used to evaluate the capacity of rats to acquire spatial learning and memory in the water maze. This evaluation was conducted over 5 days, with four daily training sessions and a 30-min interval between sessions. Throughout the training sessions, a single quadrant was randomly selected as the starting point for the rat, which was subsequently placed in water. Upon successful ascent onto the hidden platform, the rat was afforded 10 s to remain there. Conversely, if the rat could not locate the platform within 120 s, appropriate guidance was provided to direct the rat towards the platform, thereby allowing it to stay there for 10 s. An image-tracking system was utilized to document the latency period of the rats in successfully locating the platform during the experiment. The spatial probe trial measured rats’ spatial memory retention to find the platform after learning. On the 6th day of the experiment, the hidden platform was extracted from the water, and the rat was submerged with its orientation directed towards the pool wall at a randomly chosen entry point. To assess its performance, the number of crossings of the platform of the rat within 120 s, the time spent and distance traveled in each specific quadrant with the platform, and the time and distance percentages in each specific quadrant were measured during the test.

#### Z-score calculation of behavioral analysis

We utilized the standard z-score method to normalize the raw behavioral data. Z-scores serve as a statistical representation indicating the number of standard deviations (σ) that a given observation (X) deviates from the mean (μ) of the control group. Mathematically, the Z-score can be calculated using the formula Z = (X-μ)/σ (Guilloux et al., 2011). Many research efforts have utilized Z-scores to explore emotional behaviors in rodent models (Labots et al., 2016; Labots et al., 2018; Torrisi et al., 2021). Based on behavioral observations of rats at different time points and across sexes, Jean-Philippe Guilloux and colleagues have suggested that Z-scores of rodent emotions might be one of the most direct indicators reflective of human emotional states (Guilloux et al., 2011). The CON group served as the control group, and Z-scores were calculated for all the groups. The directionality of the scores was adjusted to ensure that an increase in the score values reflected an increase in the corresponding dimension. Z_SPT_ refers to SP, which measures the preference for sucrose. Z_EPMT_ represents the Z-score for EPMT results, including measures of time and distance in the open arms, the number of entries into the open arms, and their corresponding ratios. Z_FST_ is the Z-score of FST, which measures immobility time. Z_MWM_ indicates the Z-score for the MWM test results, including the total distance traveled, platform crossings, time and distance spent in the target quadrant, and their respective ratios. Z_depression_ = (–Z_SPT_ + Z_FST_)/2 was used to assess depression-like behaviors in rats. Z_anxiety_ = –Z_EPMT_ used to evaluate anxiety-like levels in rats. Z_cognize_ = Z_MWM_ used to assess cognitive function in rats.

#### Collection of fresh samples of Rat hippocampus, prefrontal cortex, hypothalamus tissues, and colonic content

1) Preparation: The rats were subjected to intraperitoneal administration of 1% pentobarbital sodium (0.3 mL/100 g) for anesthesia. 2) Dissection: After anesthesia, the chest and abdominal cavities of the rats were dissected to fully expose the heart. Following perfusion with physiological saline through the heart, the rats were decapitated, the rats’ brains were quickly removed from the skull and immediately placed on an ice tray. 3) Identification of Brain Regions: The three brain regions of interest were identified based on anatomical landmarks consistent with established brain atlases. The HIP was located using its distinct C-shape structure, the PFC was identified by its anterior position relative to the frontal lobe, and the HYP was located using key anatomical landmarks such as the optic chiasm at the anterior end, the mammillary bodies at the posterior end, and the hypothalamic sulci at the lateral boundaries. 4) Region Isolation: Each brain region was carefully dissected out. The tissue was then rapidly frozen using liquid nitrogen to halt any enzymatic activity. Simultaneously, colonic contents were collected. All samples were immediately placed to 2 mL EP tubes and promptly frozen using liquid nitrogen. Subsequently, the samples were securely stored at −80°C for future use.

#### DNA extraction and 16S rRNA gene sequence analysis

Total microbial genomic DNA from colonic content samples was extracted using the E.Z.N.A.^®^ Soil DNA Kit according to the protocol provided by the manufacturer’s instructions (Omega Bio-Tek, Norcross, GA, United States). To assess the quality and concentration of DNA, a combination of methods was employed, including 1.0% agarose gel electrophoresis and a NanoDrop^®^ ND-2000 spectrophotometer (Thermo Scientific, United States). Subsequently, the DNA samples were securely stored at −80°C for subsequent use. A PCR thermocycler, specifically the ABI GeneAmp^®^ 9700 model manufactured by ABI (CA, United States), was utilized to amplify the hypervariable region V3-V4 of the bacterial 16S rRNA gene. This amplification was performed using specific primer pairs 338F (5′-ACT​CCT​ACG​GGA​GGC​AGC​AG-3′) and 806R (5′-GGACTACHVGGGTWTCTAAT-3′) (Liu et al., 2016). The PCR reaction was conducted in triplicate with a reaction mixture composed of 5 × Fast Pfu buffer (4 μL), 2.5 mM dNTPs (2 μL), each primer at a concentration of 5 μM (0.8 μL), Fast Pfu polymerase (0.4 μL), template DNA (10 ng), and sufficient ddH_2_O to reach a final volume of 20 µL. Each sample was amplified in triplicate. The PCR product was extracted from a 2% agarose gel and purified using an AxyPrep DNA Gel Extraction Kit (Axygen Biosciences, Union City, CA, United States). The extraction process followed the precise guidelines provided by the manufacturer. Subsequently, the purified PCR product was quantified using Quantus™ Fluorometer (Promega, United States). The raw FASTQ files were de-multiplexed using an in-house Perl script, followed by the application of fastp version 0.19.6 (Chen S. et al., 2018) for quality filtering. The resulting files were subsequently merged using FLASH version 1.2.7 (Magoč and Salzberg, 2011).

#### Metabolite extraction and (UHPLC-MS/MS) analysis

Precise measurement of a 50 mg solid sample was conducted before proceeding with metabolite extraction. The extraction process involved using a 400 µL solution consisting of methanol and water in a ratio of 4:1 (v/v). To ensure accurate quantification, an internal reference, L-2-chlorophenylalanin, was added at a concentration of 0.02 mg/mL. The mixture was allowed to settle at −10°C and was subsequently treated using the high-throughput tissue crusher Wonbio-96c (Wanbo Biotechnology Co., LTD., Shanghai, China) at a frequency of 50 Hz for 6 min. To further enhance the extraction process, ultrasound was performed at 40 kHz for 30 min at 5°C. The samples were carefully stored at −20°C for 30 min to facilitate protein precipitation. After centrifugation for 15 min at 13,000 g and 4°C, the resulting supernatant was meticulously transferred into sample vials for subsequent LC-MS/MS analysis. As an integral step in the system conditioning and quality control (QC) protocol, a pooled QC sample was prepared by uniformly combining equal volumes of all individual samples. The QC samples underwent the same disposal and testing procedures as those used for the analytical samples. These samples, which represent the entire set of samples, were injected into the system at regular intervals (every eight samples) to ensure the stability of the analysis. The UHPLC-Q Exactive system manufactured by Thermo Fisher Scientific served as the platform for LC-MS analysis. Upon the successful completion of mass spectrometry detection, the raw data obtained from LC/MS analysis were preprocessed using the specialized software Progenesis QI, developed by Waters Corporation (Milford, USA). Subsequently, a three-dimensional data matrix in CSV format was generated and exported. To identify metabolites, extensive searches were conducted using recognized databases, such as Metlin (https://metlin.scripps.edu/), HMDB (http://www.hmdb.ca/), and the Majorbio Database.

#### Statistical analysis

The analysis of 16S rRNA gene sequence data involved calculating rarefaction curves and measuring alpha diversity, such as Chao1 richness, Good’s coverage, observed OTUs, and Shannon index. These calculations were performed using Mothur v1.30.1 (Schloss et al., 2009). To assess the similarity between microbial communities across different samples, principal coordinate analysis (PCoA) was conducted using the Vegan v2.5-3 package, with Bray-Curtis dissimilarity serving as the basis for comparison. Significant differences between sample groups were determined using the ANOSIM test. Using the Kruskal-Wallis test, along with linear discriminant analysis (LDA) effect size (LEfSe) (Segata et al., 2011) (http://huttenhower.sph.harvard.edu/LEfSe), the bacterial taxa (ranging from phylum to genera) exhibited significant variations across the various groups. The inclusion criteria for these taxa were an LDA score exceeding 2 and a *p*-value lower than 0.05.

In the analysis of metabolomics data, the R package ropls (Version 1.6.2) was employed to conduct principal component analysis (PCA) and orthogonal partial least squares-discriminant analysis (OPLS-DA). The model stability was assessed using a 7-cycle interactive validation approach. Additionally, Students’ t-tests and fold difference analysis were performed to further investigate the data. Differentially abundant metabolites were identified by evaluating the variable importance in projection (VIP) values derived from the OPLS-DA model and the *p*-value derived from Student’s t-test. Metabolites exhibiting VIP values exceeding 1 and *p*-values lower than 0.05 were considered to show significant differences. In addition, pathway enrichment analysis for the identified differentially abundant metabolites was performed using MetaboAnalyst 3.0 (http://www.metaboanalyst.ca/), whereas KEGG (http://www.kegg.jp) was used to identify the pathways associated with these metabolites.

The remaining data underwent statistical analysis using SPSS 22.0 software. Various statistical tests were used, such as one-way ANOVA, LSD, Welch test, Games-Howell test, Mauchly sphericity test, Greenhouse-Geisser correction, and Spearman’s correlation analysis. The analysis considered data normality and homogeneity of variance using relevant statistical approaches for each case. To report the results, the mean ± SEM was used, and statistical significance was established at a *p*-value below 0.05, indicating the presence of significant differences between the groups. Co-occurrence networks were constructed to delve into the internal community connections among the samples. The co-occurrence network was established to investigate the interrelationships between the different data components. A correlation coefficient greater than 0.6 or less than −0.6 and a *p*-value less than 0.05 indicate a statistically significant and robust association between two network nodes.

## Results

### QUE improves depressive-like behaviors and enhances cognitive function in CUMS rats

**FIGURE 2:**
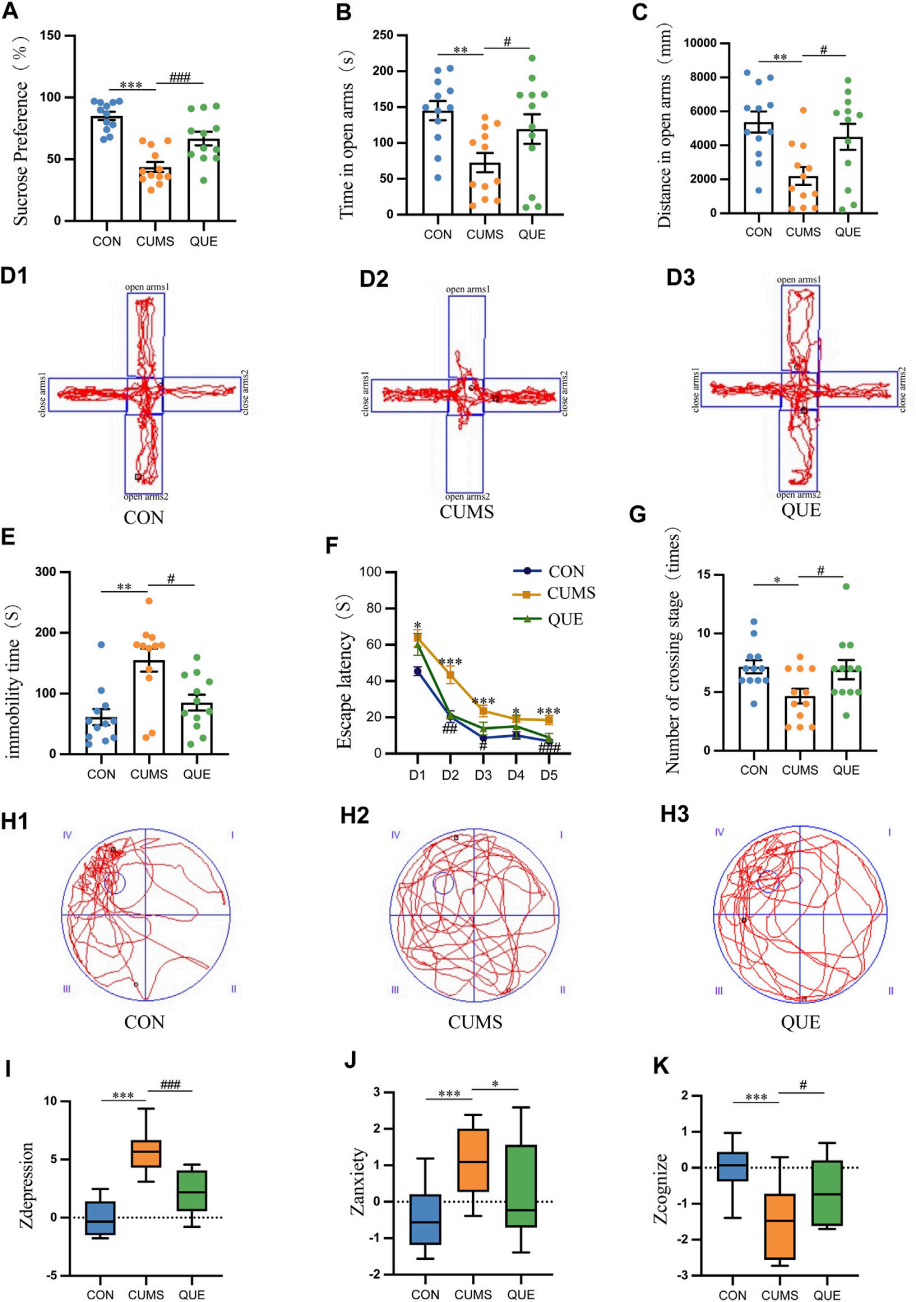
Behavioral test results in rats. **(A)** Sucrose preference (%) in SPT, *n* = 12; **(B)** Time spent in open arms (s) in EPMT, *n* = 12; **(C)** Distance traveled in open arms (mm) in EPMT, *n* = 12; **(D)** Trajectory plot in EPMT; **(E)** Immobility time (s) in FST, *n* = 12; **(F)** Escape latency (s) in MWM, *n* = 12; **(G)** Number of platform crossings in MWM; **(H)** Trajectory plot on the sixth day in MWM; **(I)** Zdepression, *n* = 12; **(J)** Zanxiety, *n* = 12; **(K)** Zcognize, *n* = 12. Data are presented as mean ± SEM. *p < 0.05, **p < 0.01, ***p < 0.001 compared to CON group; #*p* < 0.05, ##*p* < 0.01, ###*p* < 0.001 compared to CUMS group.

**FIGURE 3:**
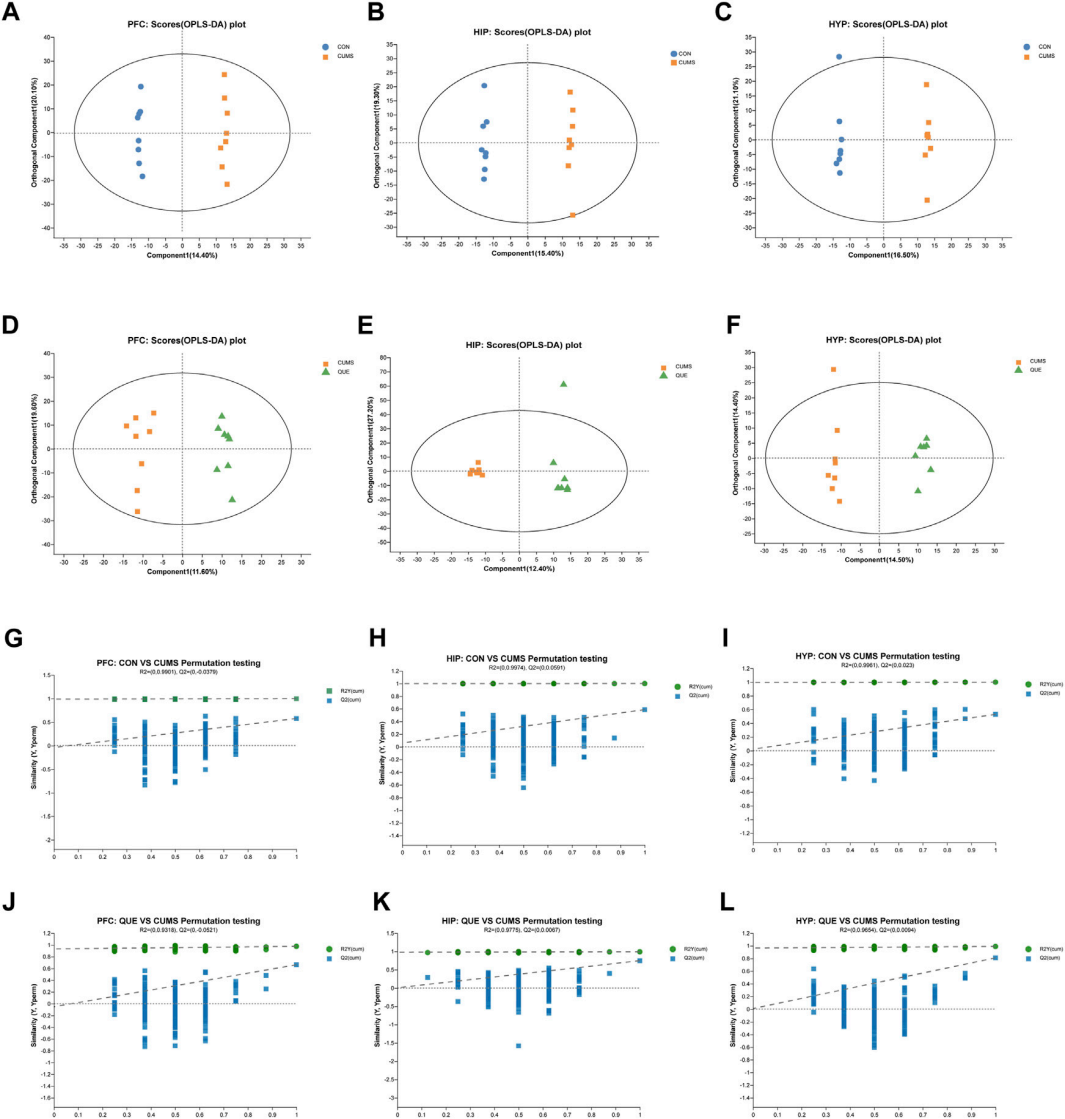
Effects of QUE on the metabolic profile of the brain in CUMS rats. **(A–F)** OPLS-DA Score Plots for CON vs. CUMS and CUMS vs. QUE in the PFC, HIP and HYP. **(G–L)** OPLS-DA replacement test (200 times) for CON vs. CUMS and CUMS vs. QUE in the PFC, HIP and HYP.

Anhedonia, anxiety-like behaviors, and depression-like behaviors in rats were evaluated using SPT, EPMT, and FST, respectively. The experimental SPT data demonstrated a notable decline in SP among rats in the CUMS group relative to that in the CON group (*p* = 1.3669E-7 < 0.001). Conversely, rats in the QUE group exhibited a notable elevation in SP compared to the CUMS group (*p* = 0.000776 < 0.001, Figure 2A). Furthermore, the EPMT results demonstrated a significant reduction in both the time spent and distance traveled in the open arms among the rats in the CUMS group compared to those in the CON group (*p* = 0.003401 < 0.01, *p* = 0.001325 < 0.01, respectively). Nonetheless, upon QUE intervention, a notable increase was observed in both the time spent and the distance traveled in the open arms (*p* = 0.049647 < 0.05, *p* = 0.015838 < 0.05, respectively), as demonstrated in Figures 3B, C, D1–D3. The FST results revealed that rats in the CUMS group exhibited a notable increase in immobility time (*p* = 0.001059 < 0.01) compared to the CON group. Conversely, compared to the CUMS group, rats in the QUE group displayed a marked reduction in immobility time (*p* = 0.018093 < 0.05, Figure 2E).

The MWM test was used to assess the learning and spatial memory functions of the rats. In the first 5 days of the place navigation test, the data did not meet the assumption of sphericity based on Mauchly’s sphericity test (*p* = 0.001017 < 0.001). As the training days increased, the escape latency to the platform decreased in all three groups (*p* = 6.9155E-16 < 0.001). During the first 5 days of the place navigation test, rats in the CUMS group took significantly more time to reach the hidden platform than those in the CON group (*p* = 3.8633E-7 < 0.001). However, after the QUE intervention, the time to reach the platform decreased significantly (*p* = 0.000046 < 0.001, Figure 2F). On the 6th day of the spatial probe test, the CUMS group displayed a substantial decrease in platform crossings (*p* = 0.013412 < 0.05). In contrast, after QUE treatment, the number of platform crossings in the QUE group significantly increased (*p* = 0.024810 < 0.05, Figures 2G, H1–H3). These results suggest that CUMS impairs spatial memory and that QUE intervention improves it in rats.

Zdepression was used to assess depression-like phenotypes in rats, Zanxiety was employed to evaluate anxiety-like phenotypes, and Zcognize was deployed to measure cognitive function. The results indicated that compared to the CON group, rats in the CUMS group displayed a significant increase in Zdepression and Zanxiety scores (*p* = 3.8744E-9 < 0.001 and *p* = 0.001302 < 0.001, respectively) and a notable decrease in Zcognize scores (*p* = 0.000434 < 0.001). After intervention with QUE, rats in the QUE group demonstrated a remarkable reduction in Zdepression and Zanxiety scores (*p* = 0.000022 < 0.001 and *p* = 0.044008 < 0.05, respectively), accompanied by a substantial increase in Zcognize scores (Figures 2I–K).

These results indicate that QUE effectively alleviates depressive-like and anxiety-like behaviors in rats, improves learning and spatial memory function, and enhances cognitive abilities.

### Impact of quercetin intervention on brain tissue metabolic profiles

To investigate the effects of CUMS and QUE intervention on the metabolic profile of rat brain tissues, we performed an untargeted metabolomic analysis of PFC, HIP, and HYP tissues. Six multivariate OPLS-DA models were constructed, with the following parameters R^2^X = 0.413, R^2^Y = 0.999, Q^2^ = 0.587 (CUMS vs. CON, HIP), R^2^X = 0.494, R^2^Y = 0.99, Q^2^ = 0.747 (CUMS vs. QUE, HIP), R^2^X = 0.435, R^2^Y = 0.999, Q^2^ = 0.53 (CUMS vs. CON, HYP), R^2^X = 0.289, R^2^Y = 0.99, Q^2^ = 0.81 (CUMS vs. QUE, HYP), R^2^X = 0.419, R^2^Y = 0.998, Q^2^ = 0.577 (CUMS vs. CON, PFC) and ^2^X = 0.312, R^2^Y = 0.976, Q^2^ = 0.662 (CUMS vs. QUE, PFC). These models demonstrated good stability and predictive abilities. The OPLS-DA model score plots (Figures 3A–F) displayed distinct separation of the metabolic profiles of the rat brain tissues (PFC, HIP, and HYP), indicating significant differences in the metabolites among the three groups. The permutation test results (Figures 3G–L) demonstrate that the models did not overfit.

**FIGURE 4:**
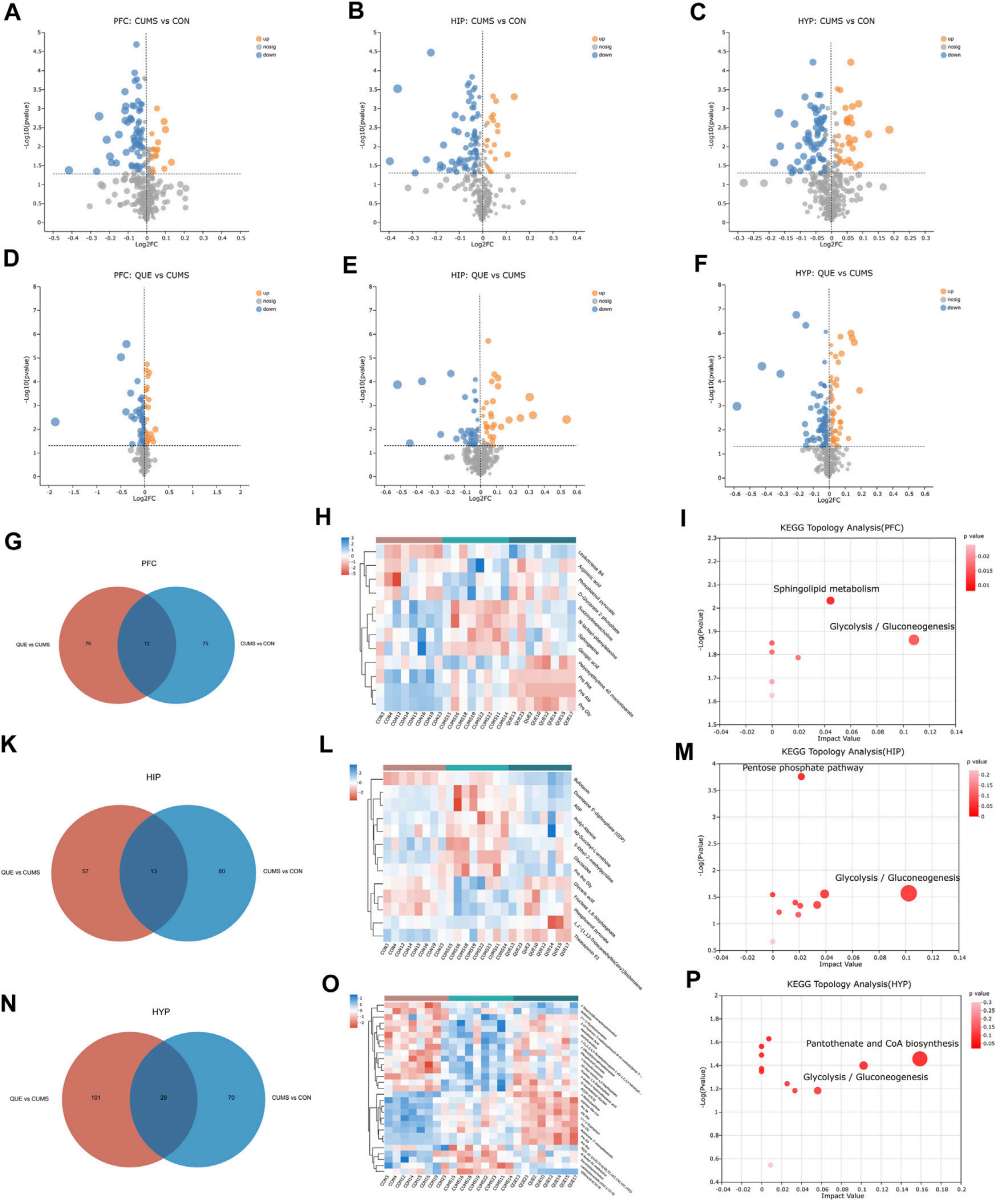
Key differential metabolites and metabolic pathways. **(A–F)** Volcano plots of differential metabolites comparing CON vs. CUMS and CUMS vs. QUE in the PFC, HIP, and HYP. **(G–I)** Venn diagrams of differential metabolites, heatmap of common differential metabolites, and KEGG topological analysis in the PFC comparing CON vs. CUMS and CUMS vs. QUE. **(K–M)** Venn diagrams of differential metabolites, heatmap of common differential metabolites, and KEGG topological analysis in the HIP comparing CON vs. CUMS and CUMS vs. QUE. **(N–P)** Venn diagrams of differential metabolites, heatmap of common differential metabolites, and KEGG topological analysis in the HYP comparing CON vs. CUMS and CUMS vs. QUE.

As revealed in the volcano plots (Figures 4A–F), differential metabolites were screened based on the VIP values and Student’s t-test *p*-values derived from the OPLS-DA model. Metabolites with VIP>1 and *p* < 0.05 were considered differentially expressed. In the PFC, 175 differential metabolites were identified, including 87 in the CON vs. CUMS comparison, 88 in the CUMS vs. QUE comparison, and 12 shared in the CUMS vs. CON and QUE vs. CUMS comparisons. Among these, eight metabolites exhibited opposite regulatory trends, including sphingosine, phosphoenol pyruvate, and D-glycerate 2-phosphate (Figures 4G,H, Supplementary Table S1). In HIP, 163 differential metabolites were identified, including 93 in the CON vs. CUMS comparison, 70 in the CUMS vs. QUE comparison, and 13 shared in the CUMS vs. CON and QUE vs. CUMS comparisons. Among these, 11 metabolites exhibited opposite regulatory trends, including glyceric acid and fructose 1,6-bisphosphate (Figures 4K,L, Supplementary Table S2). In the HYP, 229 differential metabolites were identified, including 99 in the CON vs. CUMS comparison, 130 in the CUMS vs. QUE comparison, and 29 shared in the CUMS vs. CON and QUE vs. CUMS comparisons. Among these, 18 metabolites exhibited opposite regulatory trends, including fructose 1,6-bisphosphate, pantothenic acid, and phosphoenol pyruvate (Figures 4N,O, Supplementary Table S3).

To further elucidate the potential pathways affected by quercetin, we performed metabolic pathway annotations for the common differential metabolites (metabolites altered by quercetin treatment) in the CUMS vs. CON and QUE vs. CUMS groups using the KEGG and HMDB databases. Subsequently, pathway enrichment and topology analyses were conducted using the Python software package sciPy.stats, and the main metabolic pathways involving differential metabolites were identified using Fisher’s exact test. The results revealed that two metabolic pathways, glycolysis/gluconeogenesis and sphingolipid metabolism, in the PFC (Figure 4I), two metabolic pathways, glycolysis/gluconeogenesis and pentose phosphate pathway, in the HIP (Figure 4M), and two metabolic pathways, glycolysis/gluconeogenesis and pantothenate and CoA biosynthesis, were significantly perturbed in the HYP (Figure 4P). These pathways are significant targets of quercetin’s activity, and their enhanced metabolites are the key differential metabolites (Supplementary Figure S2).

### Regulatory effects of quercetin on the gut microbiota in depression-like rats

**FIGURE 5:**
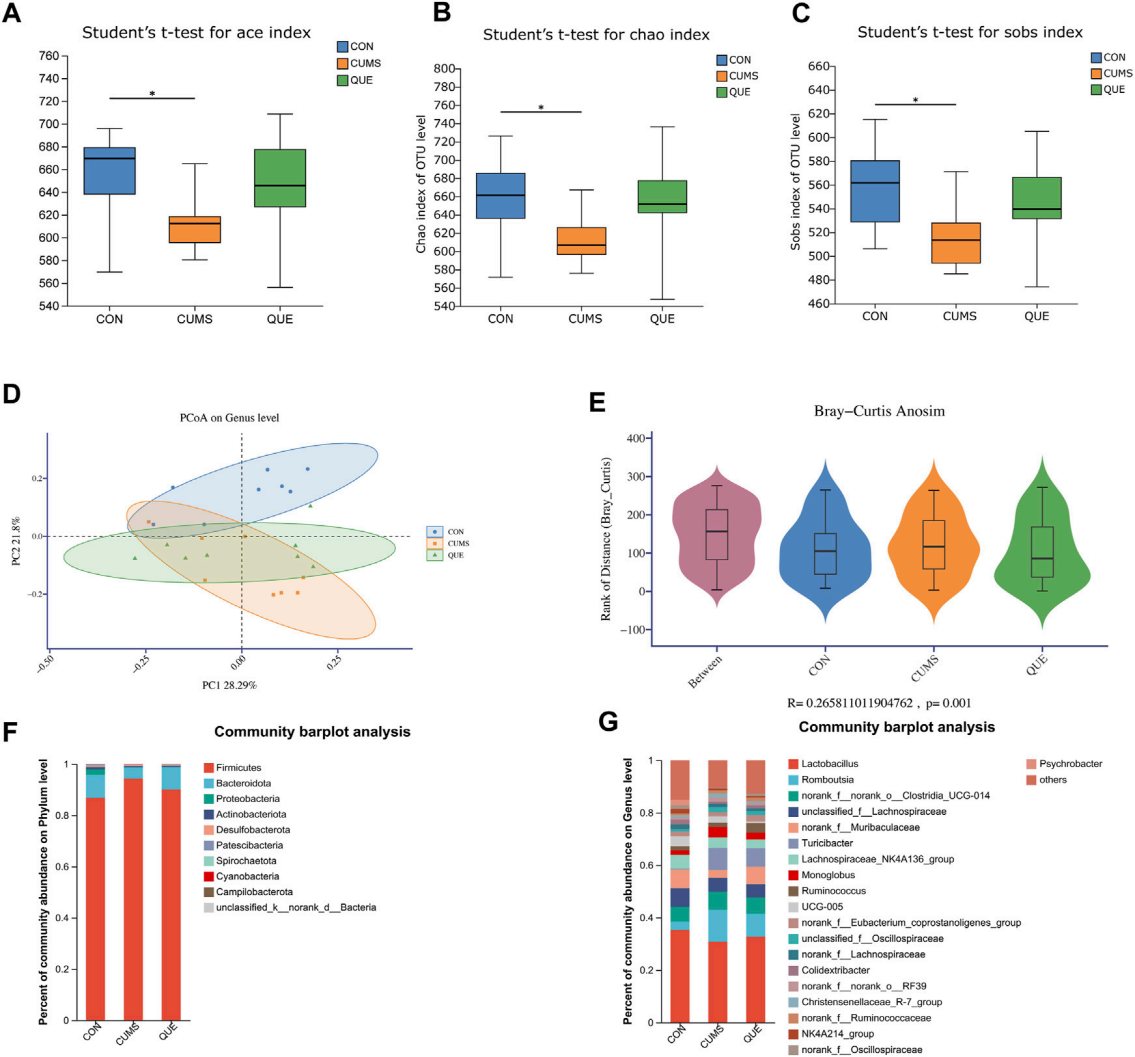
Effects of Quercetin on the Gut Microbiota of CUMS Rats. **(A)** ACE index. **(B)** Chao1 index. **(C)** Sobs index. **(D)** PCoA analysis. **(E)** In the anosim similarity analysis, R > 0 indicates larger inter-group differences than intra-group differences. **(F)** Barplot of gut microbiota abundance at the phylum level. **(G)** Barplot of gut microbiota abundance at the genus level.

We used 16S rRNA gene sequencing of colonic content samples from the three rat groups to assess gut microbiota changes after CUMS modeling and quercetin administration. After sequence optimization, QC, and rarefaction, 1,111,576 feature sequences were obtained, with an average of 46,316 effective sequences per sample. These feature sequences were classified into 1,019 Operational Taxonomic Units based on 97% sequence similarity. Alpha diversity analysis results disclosed that the ace, Chao1, and Sobs indices in the gut microbiota of CUMS rats were significantly lower than those in the CON group (Figures 5A–C), indicating that CUMS intervention significantly reduced microbial community richness. The QUE group depicted an upward trend compared with the CUMS group, but the difference was not statistically significant. We further assessed the beta diversity of the microbial community using PCoA based on Bray-Curtis dissimilarity. The PCoA results (Figure 5D) and intergroup differences test (R = 0.266, *p* < 0.05, Figure 5E) demonstrated that the gut microbiota of each group clustered significantly, and significant differences existed in the microbial composition and structure among the groups. Additionally, the clustering trend of the QUE group was similar to that of the CON group, indicating that CUMS significantly changed the gut microbial community structure and that quercetin regulated the gut microbiota of CUMS rats.

To further analyze the specific effects of the CUMS model and quercetin intervention on the rat gut microbiota, we examined the composition and abundance changes of gut microbial taxa in each group. At the phylum and genus levels, we retained the top 10 and 20 species with the highest abundance, respectively, to calculate the relative abundance and then plotted the relative abundance using a stacked bar chart (Figures 5F,G). At the phylum level, *Firmicutes*, *Bacteroidota*, and *Proteobacteria* were the dominant phyla, accounting for over 98% of the total relative abundance. Compared to the CON group, the gut of CUMS rats had more *Firmicutes*, less *Bacteroidota*, and a higher *Firmicutes/Bacteroidota* (F/B) ratio. Quercetin intervention reversed this trend (Figure 5F). At the genus level, changes in the gut microbial community of CUMS rats mainly involved a decrease in *Lactobacillus*, *norank_f__Muribaculaceae*, *Lachnospiraceae_NK4A136_group*, and *Psychrobacter*, and an increase in *Turicibacter*, *norank_f__norank_o__Clostridia_UCG-014*, *Romboutsia*, *UCG-005*, and *Monoglobus*. Quercetin intervention counteracted these changes (Figure 5G).

**FIGURE 6:**
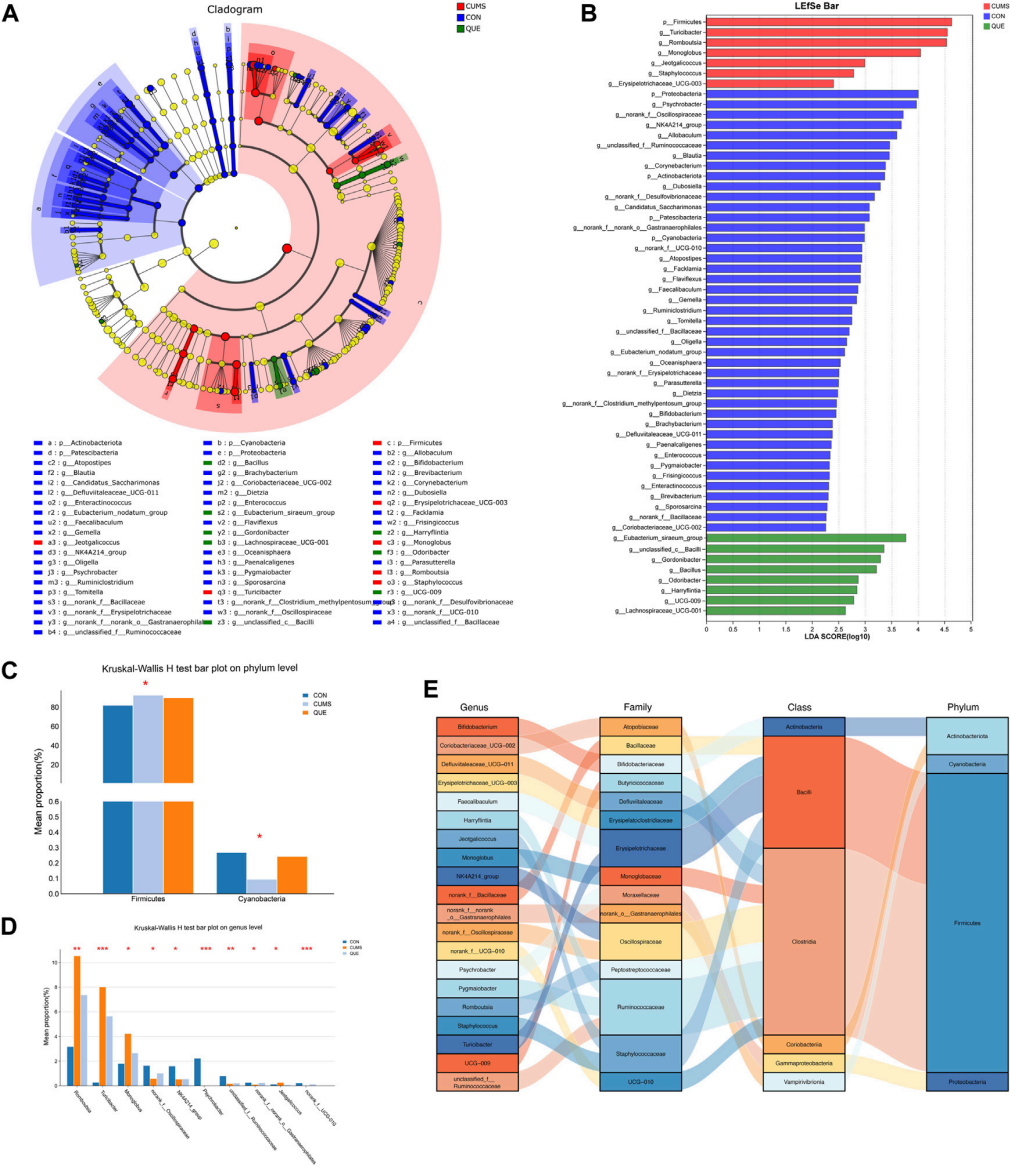
Analysis of differentially abundant bacteria in the gut microbiota of CUMS rats regulated by quercetin intervention. **(A)** Enriched microbial taxa dendrogram generated from LEfSe analysis. **(B)** Histogram depicting the distribution of differentially abundant bacteria based on LDA scores. **(C)** Significantly differentially abundant phyla with reversed abundances by quercetin intervention at the phylum level (Kruskal–Wallis H test). **(D)** Top 10 genera with reversed abundances by quercetin intervention at the genus level (Kruskal–Wallis H test). **(E)** Sankey diagram illustrating the significantly differentially abundant genera with reversed abundances by quercetin intervention at the genus level.

To identify statistically different biomarkers in the CUMS group and further determine the significant markers affected by quercetin intervention (QUE), we performed LEfSe and Kruskal–Wallis rank-sum test analyses. LEfSe analysis revealed 106 differentially abundant species from the phylum to the genus level (LDA score >2) (Supplementary Table S4, Figures 6A,B), with 53 species at the genus level and 5 species at the phylum level (Figures 6A,B). Based on the LEfSe analysis results, we further compared the differential markers among the CON, CUMS, and QUE groups using the Kruskal-Wallis rank sum test. At the phylum level, we identified five differentially abundant taxa with opposite trends in CON vs. CUMS and QUE vs. CUMS, among which *Firmicutes* and *Cyanobacteria* were statistically significant (Figure 6C, Supplementary Table S5). At the genus level, we identified 20 differentially abundant taxa with opposite trends in CON vs. CUMS and QUE vs. CUMS. Among these, *Romboutsia*, *Turicibacter*, *Monoglobus*, *Jeotgalicoccus*, *Staphylococcus*, and *Erysipelotrichaceae_UCG-003* were significantly increased in the CUMS group but decreased significantly after quercetin intervention. In contrast, *Bifidobacterium*, *Faecalibaculum*, and *Pygmaiobacter* decreased significantly in the CUMS group but increased significantly after quercetin intervention (Supplementary Table S6). The top 10 differentially abundant genera with statistical significance are displayed in Figure 6D. Furthermore, we categorized these 20 differentially abundant genera based on their taxonomy at the class, order, and phylum levels and depicted a Sankey plot (Figure 6E). Among them, 16 genera belonged to the phylum *Firmicutes*; two belong to the phylum *Actinobacteria*, one belongs to the phylum *Proteobacteria*, and one belongs to the phylum *Cyanobacteria*.

### Correlation analysis of behavioral Z scores with key differential metabolites in brain regions and gut microbiota

**FIGURE 7:**
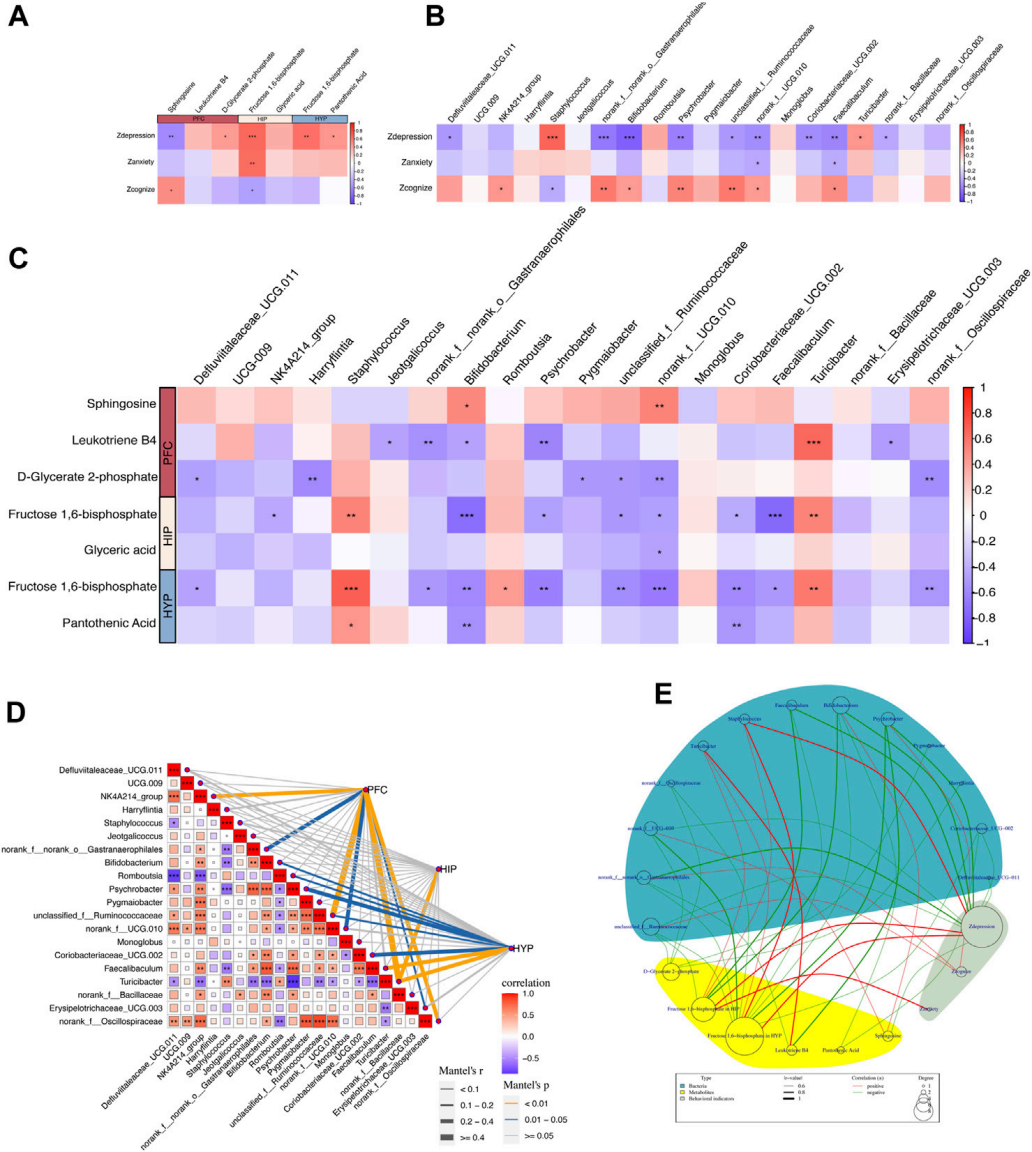
Correlation between behavioral metrics, differential metabolites, and gut differential microbiota. **(A)** Spearman’s correlation analyses between the behavioral Z scores of rats and key differential metabolites in the PFC, HIP, and HYP. **(B)** Spearman’s correlation analyses between the behavioral Z scores of rats and differential bacteria at the genus levels. **(C)** Spearman’s correlation analyses between differential bacteria at the genus level and key differential metabolites in the PFC, HIP, and HYP. **(D)** Mantel test of the correlation between differential bacteria at the genus level and key differential metabolites in the PFC, HIP, and HYP. **(E)** Network circle of the Spearman’s correlation between the behavioral Z scores of rats and key differential metabolites in the PFC, HIP, and HYP, along with differential bacteria at the genus level.

To understand how quercetin regulates the “microbiota-gut-brain” axis to treat depression, we conducted a series of correlation analyses to explore the relationships among behavioral phenotypes, brain region metabolites, and gut microbiota from three dimensions: depression-like behaviors, anxiety-like behaviors, and cognitive function in rats. First, we performed Spearman’s correlation analyses between the behavioral Z-scores of rats and key differential metabolites (Figure 7A) and differentially abundant taxa at the phylum (Supplementary Figure S1B) and genus levels (Figure 7B). Next, we conducted correlation analyses between the differentially abundant taxa at the phylum and genus levels and the key differential metabolites separately (Supplementary Figures S1C, S7C). Subsequently, Mantel tests were performed to analyze the correlation between differentially abundant taxa at the genus level and key differential metabolites (Figure 7D). Finally, we examined the relationships among the behavioral Z-scores, key differential metabolites, and differentially abundant taxa using Spearman’s correlation analyses and constructed correlation network diagrams (Figure 7E). Correlation coefficients (r) were used to assess the degree of correlation, where |r| > 0.6 indicated a strong correlation, 0.4 < |r| ≤ 0.6 indicated a moderate correlation, 0.3 < |r| ≤ 0.4 indicated a weak correlation; and |r| ≤ 0.3 indicated no correlation (Akoglu, 2018; Schober et al., 2018). We focused on results showing moderate to strong correlations (i.e., |r| > 0.4) in our study.

#### Correlation analysis of behavioral Z scores and key differential metabolites

Behavioral Z-scores were highly correlated with key differential metabolites, and several behavioral indices were highly linked to enriched differential metabolites in key pathways (Figure 7A). Specifically, Zdepression was negatively correlated with sphingosine in the PFC (r = −0.596, *p* = 0.002) and positively correlated with D-glycerate 2-phosphate in the PFC (r = 0.434, *p* = 0.033), fructose 1,6-bisphosphate in the HIP (r = 0.643, *p* = 0.0006), fructose 1,6-bisphosphate in the HYP (r = 0.612, *p* = 0.001), and pantothenic acid in the HYP (r = 0.456, *p* = 0.024). Zanxiety was positively correlated with fructose 1,6-bisphosphate in the HIP (r = 0.625, *p* = 0.001). Zcognize was positively associated with sphingosine in the PFC (r = 0.416, *p* = 0.042) and negatively correlated with D-glycerate 2-phosphate in the PFC (r = −0.445, *p* = 0.029), and fructose 1,6-bisphosphate in the HIP (r = −0.418, *p* = 0.041). These correlations imply potential associations between specific metabolites in different brain regions and behavioral phenotypes, highlighting the importance of these metabolites in modulating depression-like behaviors, anxiety-like behaviors, and cognitive function in rats.

#### Correlation analysis of behavioral Z scores and differential bacteria (phylum and genus level)

As revealed in Figure 7B, Zdepression in rats exhibited high correlations with several differential bacterial genera: Zdepression was negatively correlated with Bifidobacterium (r = −0.789, *p* = 4.46E-06) and Psychrobacter (r = −0.620, *p* = 0.001), representing nine bacterial genera; Zdepression was positively correlated with Turicibacter (r = 0.450, *p* = 0.027) and *Staphylococcus* (r = 0.663, *p* = 0.0004).

There were also correlations between Zanxiety, Zcognize, and different bacterial genera. These results suggest potential associations between specific bacterial genera and behavioral phenotypes, indicating the possible involvement of gut microbiota in modulating depression-like behaviors, anxiety-like behaviors, and cognitive function in rats.

#### Correlation analysis of key differential metabolites and differential bacteria (at the phylum and genus levels) in colonic contents

First, we performed a correlation analysis between key differential metabolites and taxa at the phylum level (Supplementary Figure S1C). The results demonstrated no significant correlation between the key differential metabolites and taxa at the phylum level. We then explored the correlation between the key differential metabolites and taxa at the genus level. The specific results of Spearman’s correlation analysis (Figure 7C) were as follows: In the PFC, sphingosine was positively correlated with Bifidobacterium (r = 0.502, *p* = 0.012) and norank_f__UCG-010 (r = 0.536, *p* = 0.006); D-glycerate 2-phosphate was negatively correlated with Harryflintia (r = −0.580, *p* = 0.002), Pygmaiobacter (r = −0.507, *p* = 0.011), unclassified_f__Ruminococcaceae (r = −0.506, *p* = 0.011), norank_f__UCG-010 (r = −0.550, *p* = 0.005), and norank_f__Oscillospiraceae (r = −0.566, *p* = 0.003). In the HIP, fructose 1,6-bisphosphate was negatively correlated with Bifidobacterium (r = −0.725, *p* = 6.02E-05), Faecalibaculum (r = −0.747, *p* = 2.74E-05), NK4A214_group (r = −0.425, *p* = 0.038), Psychrobacter (r = −0.479, *p* = 0.017), unclassified_f__Ruminococcaceae (r = −0.501, *p* = 0.012), and norank_f__UCG-010 (r = −0.473, *p* = 0.019), and positively correlated with Turicibacter (r = 0.526, *p* = 0.008) and *Staphylococcus* (r = 0.525, *p* = 0.008). Glyceric acid was negatively linked to norank_f__UCG-010 (r = −0.442, *p* = 0.030). In the HYP, fructose 1,6-bisphosphate was negatively connected to Bifidobacterium (r = −0.587, *p* = 0.002), Defluviitaleaceae_UCG-011 (r = −0.459, *p* = 0.023), norank_f__norank_o__Gastranaerophilales (r = −0.511, *p* = 0.010), Psychrobacter (r = −0.627, *p* = 0.001), unclassified_f__Ruminococcaceae (r = −0.576, *p* = 0.003), norank_f__UCG-010 (r = −0.650, *p* = 0.0005), Coriobacteriaceae_UCG-002 (r = −0.597, *p* = 0.002), Faecalibaculum (r = −0.501, *p* = 0.012), and norank_f__Oscillospiraceae (r = −0.532, *p* = 0.007); it was positively correlated with Romboutsia (r = 0.423, *p* = 0.039), Turicibacter (r = 0.566, *p* = 0.003), and *Staphylococcus* (r = 0.692, *p* = 0.0001). Pantothenic Acid was negatively correlated with Bifidobacterium (r = −0.541, *p* = 0.006) and Coriobacteriaceae_UCG-002 (r = −0.522, *p* = 0.008) and positively correlated with *Staphylococcus* (r = 0.443, *p* = 0.029). Notably, fructose 1,6-bisphosphate, a key metabolite shared by HIP and HYP, was correlated with the differential taxa (Figures 7C, E). We then performed a Mantel test to assess the overall correlation between differential taxa in the gut and brain metabolites. The results demonstrated the strongest correlation between the differential taxa and metabolites in the PFC, followed by HYP, and the weakest correlation was observed in the HIP. Turicibacter was correlated with metabolites in all three brain regions (Figure 7D). Finally, we constructed a Spearman’s correlation network graph (Figure 7E) between behavioral Z-scores, key differential metabolites in the PFC, HIP, HYP, and gut differential taxa.

## Discussion

Depression, a prevalent mental condition with a complex and heterogeneous pathogenesis, severely affects society. Traditional antidepressant drugs have certain limitations in treating depression and often have side effects. Consequently, the search for safer and more effective natural alternatives has become a hot topic of current research. Quercetin, a plant-based flavonoid, has been reported to have antidepressant properties; however, its mechanisms are unknown.

In this study, we established a Chronic Unpredictable Mild Stress (CUMS) rat model, renowned for its widespread use, reliability, and efficacy in chronic stress research in animals (Antoniuk et al., 2019; Lages et al., 2021). However, it is crucial to acknowledge that, similar to the human condition, not all animals subjected to the CUMS protocol develop depressive-like symptoms. (Castro et al., 2012; Marcolongo-Pereira et al., 2022). Some studies have introduced susceptible and resilient subgroups to explore the effects of these individual differences. Nonetheless, difficulties and controversies persist in empirically defining vulnerability *versus* resilience among animals (Willner, 2017), with CUMS stress potentially altering resilience in rats (Liu et al., 2023). Our study concentrated on the general modulatory effects of quercetin on depressive-like phenotypes induced by CUMS, without making specific distinctions between individual resilience and susceptibility. Further research is necessary to elucidate the specific manifestations in, and differences between, susceptible and resilient rats. We conducted metabolomics analysis of three brain regions (PFC, HIP, and HYP) and 16s rRNA sequencing analysis of the colon contents to assess the effect of quercetin on depression-like behaviors in rats. Our findings indicate that quercetin may be involved in reshaping gut microbiota, regulating brain metabolism, and thereby ameliorating depressive behaviors in rats (Supplementary Figure S2).

Increasing evidence suggests a close association between carbohydrate metabolism and depression, with abnormalities in carbohydrate metabolism being observed in the PFC (Baxter et al., 1989; Kennedy et al., 2001; Hennings et al., 2012; Li et al., 2015; Yao et al., 2023), HIP (Cherix et al., 2022), and HYP (Al-Massadi et al., 2021) of depression patients or depression-like animal models. This study found that quercetin may ameliorate depression-like behaviors in CUMS rats by modulating glycolysis/gluconeogenesis and the pentose phosphate pathway. Notably, quercetin strongly altered the glycolysis/gluconeogenesis pathway in all three brain areas. D-glycerate 2-phosphate was discovered as a critical differential metabolite in quercetin’s modulation of glycolysis/gluconeogenesis in the PFC. D-glycerate 2-phosphate is an intermediate product of glycolysis that, under the catalysis of enolase, can be converted into phosphoenolpyruvate and subsequently transformed into pyruvate by pyruvate kinase, leading to ATP production and playing a crucial regulatory role in depression (Ma et al., 2018; Lin et al., 2022). In our study, the level of D-glycerate 2-phosphate was significantly elevated in CUMS rats, and quercetin intervention significantly reduced its level, suggesting that CUMS might disrupt its breakdown, causing abnormal glycolysis/gluconeogenesis and subsequent depression-like behaviors. These findings were supported by the correlation analysis (Figure 7A), which demonstrated a positive correlation between D-glycerate 2-phosphate and Zdepression and a negative correlation between D-glycerate 2-phosphate and Zcognition. In HIP and HYP, fructose 1,6-bisphosphate was identified as a key differential metabolite in quercetin’s regulation of glycolysis/gluconeogenesis. In glycolysis, fructose 1,6-bisphosphate is an intermediate product that aldolase can cleave into dihydroxyacetone phosphate and glyceraldehyde 3-phosphate, which contribute to the onset and treatment of depression (Birkmayer, 1996; Demarin et al., 2004; Ma et al., 2018; Wang et al., 2021; Cho et al., 2022; Lin et al., 2022). We observed a significant increase in fructose 1,6-bisphosphate levels in the HIP and HYP of CUMS rats, which was reversed by quercetin intervention. Moreover, a strong positive correlation was observed between fructose 1,6-bisphosphate and Zdepression (Figure 7A), indicating that CUMS might induce the abnormal cleavage of fructose 1,6-bisphosphate, leading to disrupted glycolysis/gluconeogenesis and subsequent depression-like behaviors. Quercetin’s regulation of fructose 1,6-bisphosphate cleavage improved abnormal glycolysis/gluconeogenesis and alleviated depression-like behaviors. Furthermore, the pentose phosphate pathway in HIP is affected by quercetin, with glycerate being a key differential metabolite. Glycerate can be converted into 2-phosphoglycerate through the action of glycerate 2-kinase, and 2-phosphoglycerate is a key compound involved in glycolysis. Through catalysis by various enzymes, 2-phosphoglycerate completes glycolysis and produces ATP, thus playing a crucial role in regulating depression (Ma et al., 2018; Cho et al., 2022; Lin et al., 2022). Quercetin significantly lowered D-glycerate 2-phosphate in the PFC, fructose 1,6-bisphosphate and glycerate in the HIP, and fructose 1,6-bisphosphate in the HYP, improving carbohydrate metabolism and alleviating depression-like behaviors.

The lipid composition of the brain may affect perception and emotional behavior, potentially causing depression and anxiety disorders (Adibhatla and Hatcher, 2008; Yadav and Tiwari, 2014; Kornhuber et al., 2015). We discovered the potential regulatory effects of quercetin on brain lipid metabolism in CUMS rats, focusing on the PFC sphingolipid metabolism pathway, in which sphingosine is a significant differential metabolite. When correlated with behavioral indicators in rats, an elevation in sphingosine levels in the PFC was associated with reduced depression-like behaviors and improved cognitive function (Figure 7A), signifying its high relevance to depression-like behaviors. Sphingosine is a crucial intermediate in the sphingolipid metabolic pathway that links ceramides and 1-phosphosphingosine. Ceramidase degrades neuroceramides into sphingosine, which sphingosine kinases phosphorylate into 1-phosphosphingosine. Recent studies have highlighted the importance of neuroceramides and 1-phosphosphingosine in brain health (van Kruining et al., 2020). Numerous studies have reported the involvement of 1-phosphosphingosine in various neurological and psychiatric disorders, including Alzheimer’s disease (He et al., 2010; Asle-Rousta et al., 2013; van Kruining et al., 2020), depression, and anxiety (Jang et al., 2011). In our study, the CUMS group exhibited a significant reduction in sphingosine levels in the PFC, indicating abnormal sphingolipid metabolism. However, quercetin administration substantially increased sphingosine levels in the PFC, further regulating neuroceramides and 1-phosphosphingosine levels, ultimately improving sphingolipid metabolism and alleviating depression-like behaviors.

Furthermore, quercetin reduced the levels of pantothenic acid in the HYP of CUMS rats, thereby modulating the biosynthesis pathway of pantothenic acid and coenzyme A (CoA). Pantothenic acid is a universal precursor of CoA, an essential cofactor in glucose, lipid, and protein metabolism (Leonardi et al., 2005; Jonczyk et al., 2008). There have been no direct reports on the association between pantothenic acid, CoA, and psychiatric disorders. However, our findings suggest that quercetin may indirectly ameliorate depression-like phenotypes by lowering hypothalamic pantothenic acid levels and improving pantothenic acid and CoA biosynthetic metabolism.

It is well known that the stability of the gut microbiota is crucial for maintaining host health, and gut dysbiosis is an important pathogenic factor in depression (Bosch et al., 2022; Radjabzadeh et al., 2022). In our study, we analyzed the changes in the gut microbiota in response to quercetin treatment in CUMS rats and the associations between gut microbiota alterations and brain metabolism changes to explore the potential mechanism by which quercetin exerts its therapeutic effects on depression-like phenotypes through the microbiota-gut-brain axis. We found that the α-diversity index of the gut microbiota in CUMS rats was significantly reduced, and quercetin treatment reversed this change (Figures 5A–C). β-Diversity analysis displayed that the microbial structure after quercetin intervention resembled that of the CON group (Figures 5D,E). These results indicate that in CUMS rats, quercetin can ameliorate gut microbiota dysbiosis and restores stability. The *Firmicutes/Bacteroidetes (F/B)* ratio, comprising the *Firmicutes* and *Bacteroidetes* phylum, affects gut microbiota homeostasis. In our study, the *Firmicutes* phylum in the colonic contents of rats significantly increased after CUMS stress, resulting in an elevated *F/B* ratio. However, quercetin intervention led to a marked decrease in the *Firmicutes* phylum and a subsequent reduction in the *F/B* ratio. The relationship between the *F/B* ratio and depression remains controversial due to the heterogeneity and complexity of depression (Jiang et al., 2015; Chen Z. et al., 2018; Barandouzi et al., 2020). Nevertheless, there is a wealth of reliable evidence that supports our findings. Jeffery et al. reported that the *F/B* ratio was significantly increased in patients with irritable bowel syndrome (IBS), obesity, and depression, accompanied by anxiety and depression-like behaviors (Jeffery et al., 2012). De Palma et al. demonstrated that transplantation of high *F/B* ratio feces induced anxiety and depression-like behaviors in mice (De Palma et al., 2017). Furthermore, at the genus level, quercetin reversed the changes in the abundance of 20 gut microbial genera in CUMS rats, which were highly correlated with depression-like phenotypes in rats (Figure 7B). Among them, 16 genera belong to the *Firmicutes* phylum, including *Romboutsia*, *Turicibacter*, and *Faecalibaculum*. Previous studies have linked *Romboutsia* and *Turicibacter* to circulating inflammation (IL-1β) and behavioral outcomes (hypersomnia and anxiety-like behavior), indicating that an increase in *Romboutsia* and *Turicibacter* exacerbates inflammation and anxiety-like behavior (Grant et al., 2021). *Faecalibaculum* produces short-chain fatty acids that are vital for improving cognitive function (D’Amato et al., 2020). Consistent with these findings, we found that quercetin significantly reduced *Romboutsia* and *Turicibacter* in the gut of CUMS rats and significantly increased *Faecalibaculum*, which reduced depression-like behaviors. Correlation analysis revealed that *Romboutsia* was positively correlated with 1,6-fructose diphosphate in HYP, *Turicibacter* was positively correlated with 1,6-fructose diphosphate in HIP and HYP, and *Faecalibaculum* was negatively correlated with 1,6-fructose diphosphate in HIP and HYP (Figure 7C). This indicates that *Romboutsia*, *Turicibacter*, and *Faecalibaculum*, regulated by quercetin, may further affect brain glucose metabolism, thereby potentially alleviating depression-like behaviors. *Bifidobacterium*, a member of the *Actinobacteria* phylum, is a well-studied probiotic known to improve depression-like behaviors in hosts (Ohland et al., 2013; Abildgaard et al., 2017a; Abildgaard et al., 2017b; Pinto-Sanchez et al., 2017). Our study found that quercetin increased *Bifidobacterium* abundance in CUMS rats, thereby potentially improving the depressive symptoms. Notably, *Bifidobacterium* was positively correlated with sphingosine in the PFC and negatively correlated with 1,6-fructose diphosphate and pantothenic acid in HIP and HYP. This suggests that quercetin may regulate the PFC sphingosine metabolism and the sugar metabolism in the HIP and HYP by modulating the *Bifidobacterium* in the gut, thereby potentially improving depression-like phenotypes. Moreover, 17 other genera, including *norank_f__UCG-010* and *Psychrobacter*, were identified as the key targets of quercetin in the gut microbiota of CUMS rats, particularly due to their strong correlation with brain metabolites (Figure 7C). However, these genera have not been thoroughly associated with depression, and their mechanisms require further studies.

In summary, our study provides new findings supporting the role of quercetin in alleviating depression-like phenotypes via multiple mechanisms. 1) Quercetin may regulate key metabolic pathways in the brain regions (PFC, HIP, and HYP) to alleviate depression-like behaviors induced by CUMS. 2) Quercetin may restore the gut microbiota balance by targeting specific bacteria (*Romboutsia*, *Turicibacter*, *Faecalibaculum*, and *Bifidobacterium*), contributing to its antidepressant effects. 3) Due to the correlation between behavioral changes, metabolite levels, and bacterial abundance in rats, quercetin may act on the microbiota-gut-brain axis to intervene in depression-like behaviors. Our findings provide information on the antidepressant potential and its possible mode of action, emphasizing the gut-brain relationship in depression treatment.

## Limitations

1) We focused on the general effects of quercetin on the depressive-like phenotypes induced by CUMS in animal models, without making specific distinctions between individual resilience and susceptibility. 2) Our study utilized male rats, a choice that may not fully represent the female population. Given that depression is more prevalent in female population, it is essential that future research further incorporates considerations of gender differences. 3) This study identified potential metabolic pathways targeted by quercetin; however, the specific mechanisms of action remain unclear. Further experimental research is required to investigate the detailed metabolic processes involved in these pathways. 4) We discovered several microbial targets that may be influenced by quercetin, including *bacteria like norank_f__UCG-010*, *Psychrobacter*, *unclassified_f__Ruminococcaceae*, and *Staphylococcus*. However, limited data are available for many of these targets, necessitating further exploration and in-depth studies. 5) Our results suggested an association between quercetin and alterations in the gut microbiota and metabolic profile; however, they did not definitively prove causation. Further research, including longitudinal studies and experimental interventions, is needed to elucidate the potential causal mechanisms underlying these observations. 6) Our attempt to explore the targets of quercetin in the microbiota-gut-brain axis is a step towards understanding the complex and multifaceted mechanisms of this axis. However, further research is needed to understand the effects of quercetin on the microbiota-gut-brain axis.

## Data Availability

The original contributions presented in the study are publicly available. The metabolomics data have been deposited in the MetaboLights database (https://www.ebi.ac.uk/metabolights/MTBLS9544), and the 16S rRNA sequencing data are available in the NCBI BioProject database (https://www.ncbi.nlm.nih.gov/bioproject/PRJNA1077291).
