# Supplementary figures and images for: Quercetin reshapes gut microbiota homeostasis and modulates brain metabolic profile to regulate depression-like behaviors induced by CUMS in rats
Source: Front Pharmacol. 2024 Mar 26;15:1362464. doi: 10.3389/fphar.2024.1362464 (PMC11002179; doi:10.3389/fphar.2024.1362464)

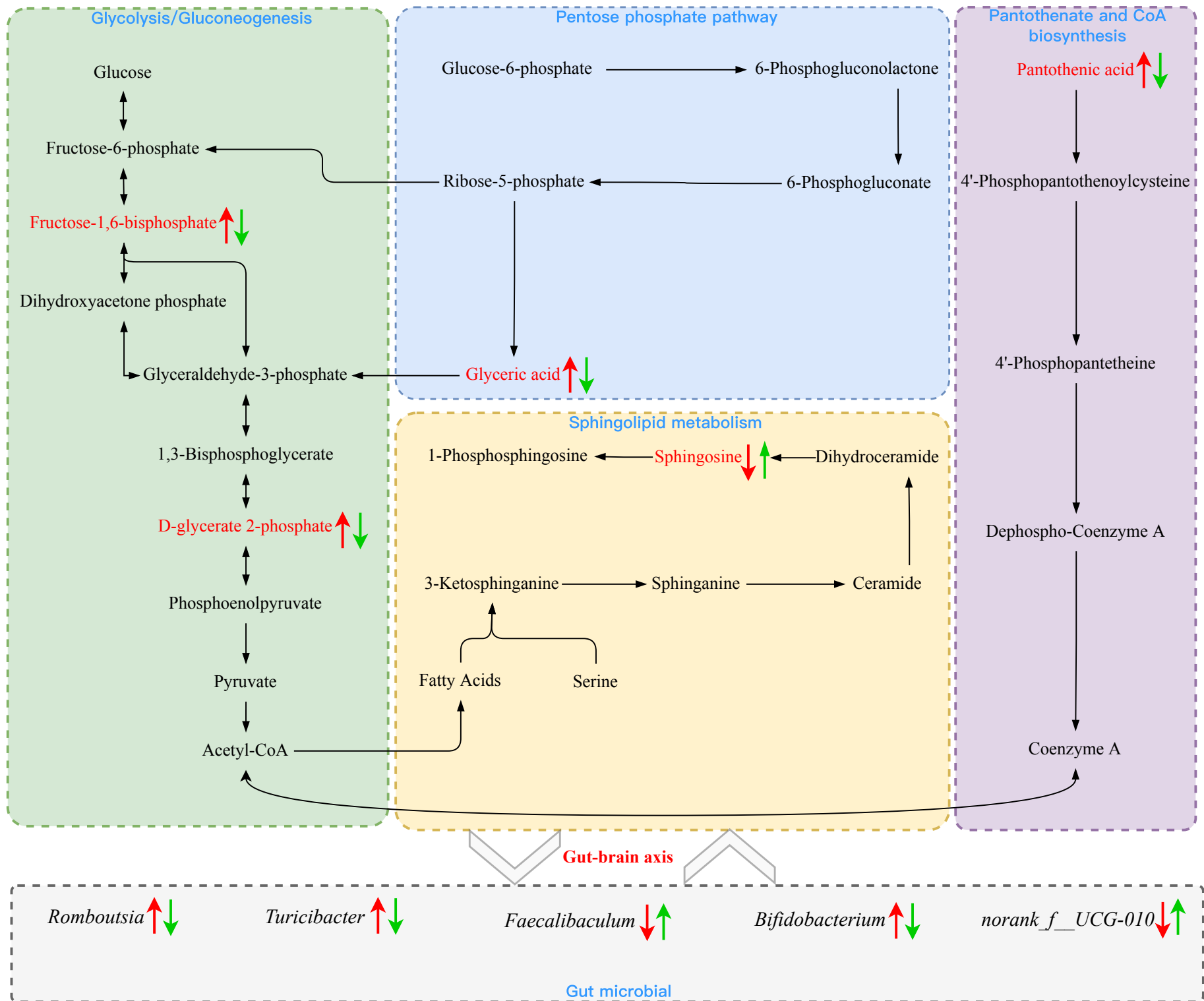

Supplement: Supplementary file 1 [file Image2.PDF]

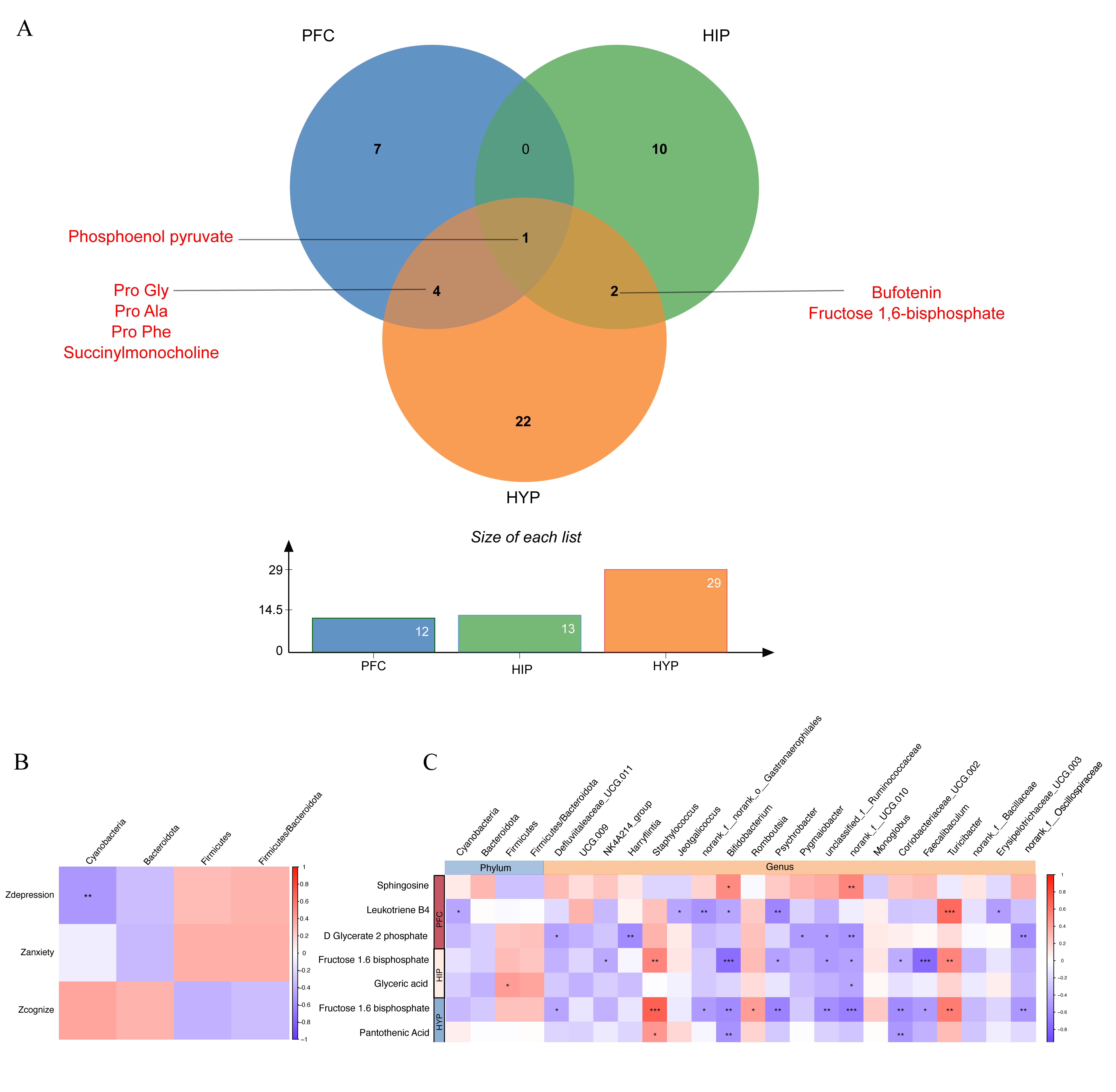

Supplement: Supplementary file 3 [file Image1.PNG]
